# Molecular characterization of exon 3 of caprine myostatin gene in Marwari goat

**DOI:** 10.14202/vetworld.2016.676-679

**Published:** 2016-06-30

**Authors:** Jai Prakash Khichar, Gyan Chand Gahlot, Vijay Kumar Agrawal, Kiran, Ajay Singh Dewna, Prakash, Mohammad Ashraf

**Affiliations:** 1 Molecular Genetics Laboratory, Department of Animal Genetics and Breeding, College of Veterinary & Animal Science, Rajasthan University of Veterinary and Animal Science, Bikaner, Rajasthan, India; 2 Department of Clinical Veterinary Medicine, Ethics and Jurisprudence, College of Veterinary and Animal Science, Bikaner, Rajasthan, India

**Keywords:** exon 3, Marwari goat, myostatin gene, polymerase chain reaction-single strand conformation polymorphism

## Abstract

**Aim:**

To estimate genetic variability in exon 3 of caprine myostatin gene in Marwari goats.

**Materials and Methods:**

A total of 120 blood samples from unrelated Marwari goats were randomly collected from different villages of Bikaner (Rajasthan), India. Genomic DNA was extracted from whole blood using blood DNA isolation kit (Himedia Ltd.) as per manufacturer’s protocol. The quality of extracted genomic DNA was checked on 0.8% agarose gel. Specifically designed a primer set for caprine myostatin (*MSTN*) gene (Genebank accession no. DQ167575) was used to amplify the exon 3 region of *MSTN* gene in Marwari goat. The genetic variability in exon 3 of *MSTN* gene in Marwari goat was assessed on 8% polyacrylamide gel electrophoresis to detect single strand conformation polymorphism (SSCP) pattern.

**Results:**

The exon 3 of *MSTN* gene in Marwari goat showed two types of conformation patterns on 8% polyacrylamide gel. One of the patterns showed only two bands and was considered as genotype AA, whereas another pattern having an extra band was designated as genotype AB. The frequencies of AA and AB genotype for exon 3 region of *MSTN* gene were calculated as 0.90 and 0.10, respectively.

**Conclusion:**

Low level of polymorphism was observed at exon 3 region of *MSTN* gene in Marwari goat through SSCP analysis. This information could be utilized in future breeding plan to exploit the unique characteristics of Marwari goat of Rajasthan.

## Introduction

Marwari goat is a major meat breed of Rajasthan (India) that is well-adapted to the arid environment and can tolerate higher salt loads and requires less water. The breed is known for its faster growth and breeding efficiency [1]. The unique characteristics of this breed require its molecular characterization for growth traits. Myostatin (*MSTN*) or growth and differentiation factor 8 is a member of the transforming growth factor-β superfamily that plays an important role in the regulation of muscle growth and meat quality [2]. The three exons and two introns of *MSTN* gene encode a glycoprotein that is expressed widely in skeletal muscles [3]. *MSTN* gene plays a critical role in myogenic differentiation and results in failure of myoblast to differentiate into myotubes through inhibition of MyoD activity and expression via Smad3 [4]. The altered gene structure could affect the composition of muscle fiber and thereby muscle weight [5]. Dramatic muscularity and a “double-muscling” phenomenon had been observed in many livestock species following mutation in the *MSTN* gene that inactivated its expression or produced a non-functional protein [6]. A number of studies in pigs, cattle [7,8], and sheep have detected the role of *MSTN* gene in muscular development [9]. However, a similar investigation about the properties of the *MSTN* gene in goat is limited [10].

Polymerase chain reaction (PCR) based single-strand conformation polymorphism (SSCP) technique has emerged as a simple, efficient, and powerful tool to detect small genetic variation in amplified product even at single base pair. Routine electrophoresis could not detect change in single nucleotide in a particular sequence of double-stranded DNA as the physical properties of the double strands are almost identical for both alleles. In SSCP technique, the double-stranded DNA after denaturation undergoes three-dimensional folding and may assume a unique conformational state based on its DNA sequence. The difference in shape between two single-stranded DNA strands with different sequences can cause them to migrate differently on polyacrylamide gel, even though the number of nucleotides is same, which is, in fact, an application of SSCP [11].

Therefore, the present study was carried out to detect genetic variation in exon 3 of *MSTN* gene in Marwari goat using the PCR-SSCP technique.

## Materials and Methods

### Ethical approval

All essential procedures of sample collection were performed strictly as specified by Institutional Ethical Committee with minimal stress to animals.

### Experimental animals

Random blood samples (n=120) were collected from unrelated Marwari goats from the field units of AICRP on Goat Improvement Project functioning at the Department of Animal Genetics and Breeding, College of Veterinary and Animal Science, Bikaner.

### Location of study

The study was conducted at the Molecular Genetics Laboratory of Department of Animal Genetics and Breeding, College of Veterinary and Animal Science, Bikaner, Rajasthan, India.

### Collection of blood samples

About 2-3 ml of venous blood was collected from jugular vein of each animal in EDTA-containing vacutainer tubes under sterile conditions. The vials were shaken gently after blood collection to facilitate thorough mixing and then kept in ice box containing ice packs. The samples were transported to the laboratory immediately and processed on the same day to extract genomic DNA.

### Extraction of genomic DNA and PCR amplification

**Figure-1:**
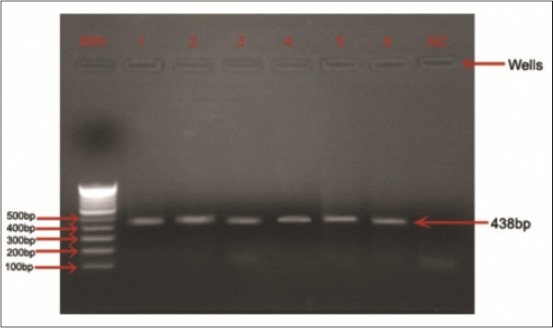
Amplification of myostatin (exon 3) gene using the specific primer of polymerase chain reaction in Marwari goats visualize under ultraviolet illuminator stained with ethidium bromide. MW: Molecular weight Marker, Wells: 1-6 myostatin (exon 3) gene 438 bp (Marwari goat), NC: Negative control.

Genomic DNA was extracted from whole blood by Blood Genomic DNA Purification Kit (HI Media Pvt. Ltd.). The exon 3 of *MSTN* gene was amplified using one set of primer designed from caprine *MSTN* gene sequence (Genebank accession no. DQ167575). The PCR reactions were carried out in a final volume of 25 µL containing 80 ng of DNA template, 0.1 µM of each primer, 10.0 mM dNTPs, 2.5 U of *Taq* DNA polymerase (Promega, Madison, USA), 5 µL ×5 reaction buffer, 1.5 mM MgCl_2_, and 11 µL ddH_2_O. An initial denaturation at 95°C for 5 min was carried out and followed by 40 cycles of denaturation at 94°C for 45 s, annealing for 45 s (Table-1), and extension at 72°C for 60 s. A final extension of 10 min was carried out at 72°C. The PCR amplified products were checked on 1.2% agarose gel (Figure-1).

**Table-1:** Primer sequences used to amplify myostatin gene.

Gene	Forward and reverse sequence (5’ to 3’)	Expected product size	Annealing temp (°C)
*MSTN* (Exon 3)	F-5’ TTTCTTTAATAATGACTCCCTGCG-3’ R-5’ TCTACTACCATGCCTGGAATCTTC-3”	438 bp	55°

### Detection of genetic variation

The polymorphism in exon 3 of *MSTN* gene was detected by SSCP analysis. Aliquot of 5 µL PCR products were mixed with 5 µL of denaturing ×2 gel loading dye and heated for 8 min at 95°C and finally chilled on ice for 7 min. 8% polyacrylamide gel electrophoresis (PAGE) was carried out with denatured amplified product to identify the different genotypic patterns. The gels were stained with ethidium bromide, analyzed under ultraviolet light, and documented by UVP gel-documentation system.

## Results and Discussion

**Figure-2:**
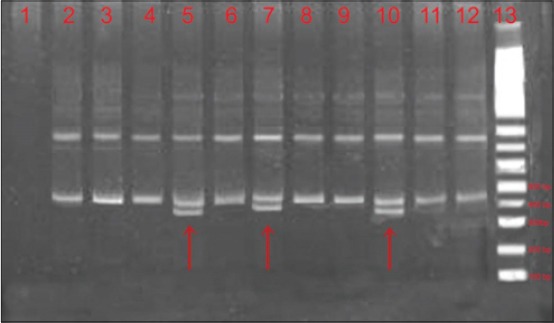
8% polyacrylamide gel electrophoresis and single strand conformation polymorphism (SSCP) analysis of polymerase chain reaction (PCR) amplified product of exon 3. Lane 1: Control blank, Lane 2-12: SSCP analysis of different samples. Arrow indicating a PCR product producing an extra lower band, Lane 13: DNA marker.

The exon 3 of *MSTN* gene in Marwari goat revealed two different conformation patterns on polyacrylamide gel (Figure-2). One of the patterns considered as genotype “AB” was observed in 12 individuals, whereas another pattern was designated as “AA” genotype and was found in most of the animals studied (n=108). The pattern AA revealed only two bands and was considered as homozygote, whereas the other pattern AB that showed three bands was designated as heterozygote. The genotypic frequencies of AA and AB genotype in the present study were found to be 0.90 and 0.10, respectively. The frequency of allele A was found to be highest (0.95) for exon 3 of *MSTN* gene in Marwari goat (Table-2). However, the present study could not detect the second homozygote.

**Table-2:** Depiction of gene and genotype frequency of Exon 3 of *MSTN* gene by SSCP.

Gene	Genotype frequency			Gene frequency	
	AA	AB	BB	A	B
Exon 3	0.90 (n=108)	0.10 (n=12)	-	0.95	0.05

SSCP=Single strand conformation polymorphism

The growth traits of animals are regulated by polygenes and are always of primary concern during breeding for determining the animal’s economical value [4]. Identification of genetic markers for growth traits is the initial and critical step for the establishment of marker-assisted selection system [12]. The detection of genetic variations in DNA sequence could help in genetic characterization of livestock breeds and may help in identification of possible hybridization events or past evolutionary trends. The change in DNA sequence of exon region of genes may lead to change in amino acid sequences and ultimately the structure of expressed protein. The polymorphism in DNA could be linked to economic traits in many livestock species [13].

A major gene model suggests that few candidate genes are responsible for relatively large proportion of genetic variation and could be utilized as marker [14]. Such major genes are usually involved in the biology of a trait and have an effect on the physiological pathway, metabolism, and expression of phenotypes. Growth hormone (GH), GH receptor, insulin-like growth factor I, leptin, caprine pituitary-specific transcription factor-1, caprine myostatin (*MSTN*), and bone morphogenetic protein genes are few candidate genes that play an important role in bone formation, birth weight, weaning weight, body condition, and muscle growth.

Myostatin protein acts as a negative regulator of skeletal muscle growth and keeps the skeletal musculature within appropriate proportions [15]. The protein inhibits terminal differentiation of myoblasts and the proliferation of myogenic cells [16,17]. Investigations of myostatin developmental expression in bovine skeletal muscles [18] and its function in myogenesis and adipogenesis had shown the relationship of myostatin expression with animal growth [19-23] observed the association of an inactive *MSTN* allele with higher birth weights and yearling weights in Piedmontese crossbred cattle. A double-muscling phenomenon was observed in Belgian Blue and Piedmontese cattle following mutation in coding region of *MSTN* gene.

The polymorphisms of *MSTN* gene were shown to have significant difference among goat breeds. It was reported that TTTTA deletion phenomenon occurred in *MSTN* gene was unique for goats when compared with sheep, cattle, water buffalo, domestic yak, pigs, and humans [6,24-26] found an important effect of a 5-bp deletion on early body weight and size of a goat.

The present study focused on identification of possible genetic variants in exon 3 of *MSTN* gene in Marwari goat keeping in view the scanty information available for caprine *MSTN* gene. The present study in Marwari goat revealed low genetic diversity in exon 3 of *MSTN* gene which is in agreement with the similar study conducted in Saanen and Boer × Guanzhong goats by An *et al*. [27], who also observed one homozygote and a heterozygote genotypic pattern revealed by SSCP in exon 3 of caprine *MSTN* gene. However, a similar type of study in Sanjabi and Zel sheep by Soufy *et al*. [28] and Dehnavi *et al*. [29] revealed a monomorphic genotypic pattern in exon 3 of *MSTN* gene that suggests the fixation of this locus in sheep. The results of the present study indicate that low level of polymorphism still exists in this meat type of breed. The inconsistency in the results may be ascribed to species and breed differences, population and sampling size, environmental factors, mating strategies, geographical position effect, and frequency distribution of genetic variants. The present study could provide a base for further investigation of caprine *MSTN* gene in Marwari goats and other native breeds using large number of samples.

## Conclusion

The PCR-SSCP analysis of exon 3 of *MSTN* gene in Marwari goat revealed low level of polymorphism. This information could be utilized in future breeding plan to exploit the genetic potential of Marwari goat.

## Authors’ Contributions

GCG and JPK designed the work plan. JPK, K, and ASD collected and processed the blood samples. JPK, MA, and VKA carried out PCR, electrophoresis, and PAGE. GCG, JPK, and P compiled, tabulated, transformed, and analyzed the data. JPK and GCG interpreted the results. JPK, VKA, and K prepared the manuscript. All authors read and approved the final manuscript.

## References

[1] Rohilla P.P, Patel A.K (2003). Marwari goat breed of Rajasthan. Indian J. Anim. Sci.

[2] Zhang C, Liu Y, Xu D, Wen Q (2012). Polymorphisms of myostatin gene (MSTN) in four goat breeds and their effects on Boer goat growth performance. Mol. Biol. Rep.

[3] Bellinge R.H, Liberles D.A, Iaschi S.P, O’Brien P.A (2005). Myostatin and its implications on animal breeding:A review. Anim. Genet.

[4] Jin X.Y (2011). Cloning and bioinformatics analysis on myostatin (MSTN) gene of goat. China Anim. Husband Vet. Med.

[5] Chen T.T (2008). Polymorphisms of MSTN, IGFBP-3 gene and the related research with growth performance of Tian Fu goat, Sichuan agricultural university, Ya’an. Asian J. Anim. Vet. Adv.

[6] Grisolia A.B, D’Angelo G.T, PortoNeto L.R, Siqueira F (2009). Myostatin (GDF8) single nucleotide polymorphisms in Nellore cattle. Genet. Mol. Res.

[7] Walsh F.S, Celeste A.J (2005). Myostatin:A modulator of skeletal-muscle stem cells. Biochem. Soc. Trans.

[8] Fan B, Lkhagvadorj S, Cai W, Young J (2010). Identification of genetic markers associated with residual feed intake and meat quality traits in the pig. Meat Sci.

[9] Boman I.A, Klemsetsdal G, Blichfeldt T, Nafstad O (2009). A frame shift mutation in the coding region of the myostatin gene (MSTN) affects carcass conformation and fatness in Norwegian white sheep *(Ovisaries)*. Anim. Genet.

[10] Liu Z.T, Li X.L, Gong Y.F, Jin X.M (2006). Relationship between polymorphism of goat MSTN gene intron 2 and body weight. Acta Vet. Zootech. Sin.

[11] Orita M, Iwahana H, Kanazawa H, Hayashi K, Sekiya T (1989). Detection of polymorphisms of human DNA by gel electrophoresis as single-strand conformation polymorphisms. Proc. Natl. Acad. Sci. USA.

[12] Li M, Li Y, Lu L, Wang X, Gong Q, Duan C (2009). Structural, gene expression, and functional analysis of the fugu (Takifugurubripes) insulin-like growth factor binding protein-4 gene. Am. J. Physiol. Regul. Integr. Comp. Physiol.

[13] Gelderman H (1997). Investigations on inheritance of quantitative characters in animals by gene markers. Methods Theoret. Appl. Genet.

[14] Lande R (1981). The minimum number of gene contributing to qualitative variation between and within populations. Gene.

[15] Lee S.J, McPherron A.C (1999). Myostatin and the control of skeletal muscle mass. Curr. Opin. Genet. Dev.

[16] Thomas M, Langley B, Berry C, Sharma M, Kirk S, Bass J, Kambadur R (2000). Myostatin, a negative regulator of muscle growth, functions by inhibiting myoblast proliferation. J. Biol. Chem.

[17] Wiener P, Woolliams J.A, Frank-Lawale A, Ryan M, Richardson R.I, Nute G.R, Wood J.D, Homer D, Williams J.L (2009). The effects of a mutation in the myostatin gene on meat and carcass quality. Meat. Sci.

[18] Shibata M, Ohshima K, Kojima T, Muramoto T, Matsumoto K, Komatsu M, Aikawa K, Fujimura S, Kadowaki M (2003). Nucleotide sequence of myostatin gene and its developmental expression in skeletal muscles of Japanese black beef. J. Amin. Sci.

[19] Lin J, Arnold B, Della-Fera M.A, Azain M.J, Hartzell D.L, Baile C.A (2002). Myostatin knockout in mice increases myogenesis and decreases adipogenesis. Biochem. Biophys. Res. Commun.

[20] Joulia D, Bernardi H, Garandel V, Rabenoelina F, Vernus B, Cabello G (2003). Mechanisms involved in the inhibition of myoblast proliferation and differentiation by myostatin. Exp. Cell Res.

[21] Rebbapragada A, Benchabane H, Wrana J.L, Celeste A.J, Attisano L (2003). Myostatin signals through a transforming growth factor β-like signaling pathway to block adipogenesis. Mol. Cell Biol.

[22] Wagner K.R, Liu X, Chang X, Allen R.E (2005). Muscle regeneration in the prolonged absence of myostatin. Proc. Nat. Acad. Sci.

[23] Casas E, Keele J.W, Fahrenkrug S.C, Smith T.P.L, Cundiff L.V, Stone R.T (1999). Quantitative analysis of birth, weaning, and yearling weights and calving difficulty in piedmontese crossbreds segregating an inactive myostatin allele. J. Anim. Sci.

[24] Kambadur R, Sharma M, Smith T.P.L, Bass J.J (1997). Mutations in myostatin (GDF8) in double-muscled belgian blue and piedmontese cattle. Genome Res.

[25] Hadjipavlou G, Matika O, Clop A, Bishop S.C (2008). Two single nucleotide polymorphisms in the myostatin (GDF8) gene have significant association with muscle depth of commercial charollais sheep.

[26] Zhang Z.J, Ling Y.H, Wang L.J, Hang Y.F, Guo X.F, Zhang Y.H, Dingand J.P, Zhang X.R (2013). Polymorphisms of the myostatin gene (*MSTN*) and its relationship with growth traits in goat breeds. Genet. Mol. Res.

[27] An X.P, Wang J.G, Hou J.X, Zhao H.B, Bai L, Li G, Wang L.X, Liu X.Q, Xiao W.P, Song Y.X, Cao B.Y (2011). Polymorphism identification in the goat *MSTN* gene and association analysis with growth traits. Czech J. Anim. Sci.

[28] Soufy B, Mohammad A, Shojaeian M.R, Baghizadeh K, Ferasaty A, Askari S.N, Dayani O (2009). Evaluation of myostatin gene polymorphism in Sanjabi sheep by PCR-RFLP method. Anim. Sci. Res. Tabriz Univ.

[29] Dehnavi E, Azari A.M, Hasani S, Nassiry M.R, Mohajer M, Khan A.A, Shah M.L, Yousefi S (2012). Polymorphism of myostatin gene in intron 1, 2 and exon 3 and their associations with yearling weight using PCR-RFLP and PCR-SSCP techniques in Zel sheep. Biotechnol. Res. Int.

